# Supplementary figures and images for: The plasma biomarker soluble SIGLEC-1 is associated with the type I interferon transcriptional signature, ethnic background and renal disease in systemic lupus erythematosus
Source: Arthritis Res Ther. 2018 Jul 27;20:152. doi: 10.1186/s13075-018-1649-1 (PMC6062988; doi:10.1186/s13075-018-1649-1)

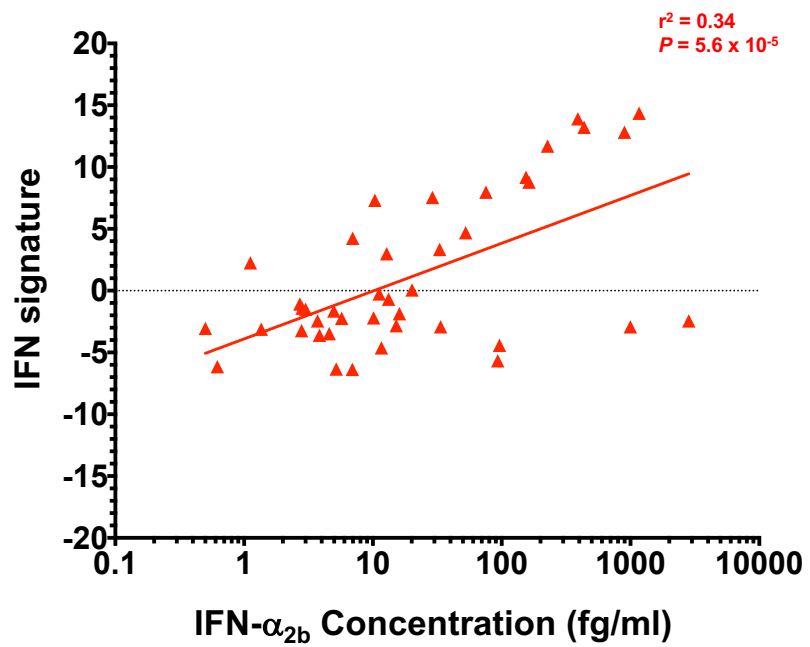

Supplement: Supplementary file 2 — Figure S1. Concentration of IFN-αb2 is associated with the IFN transcriptional signature. Data depict correlation between plasma IFN-αb2 concentration and transcriptional IFN signature in 41 SLE patients from cohort 2. (PDF 35 kb) [file 13075_2018_1649_MOESM2_ESM.pdf]

a

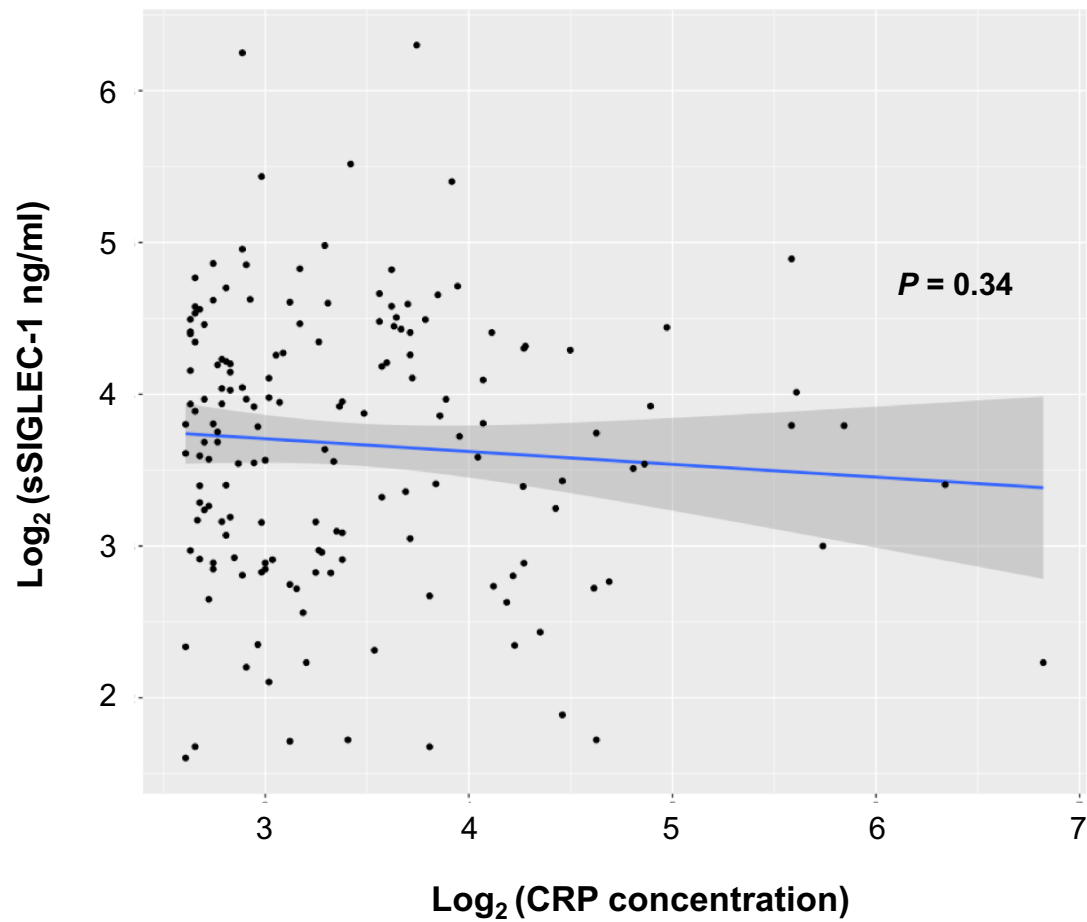

b

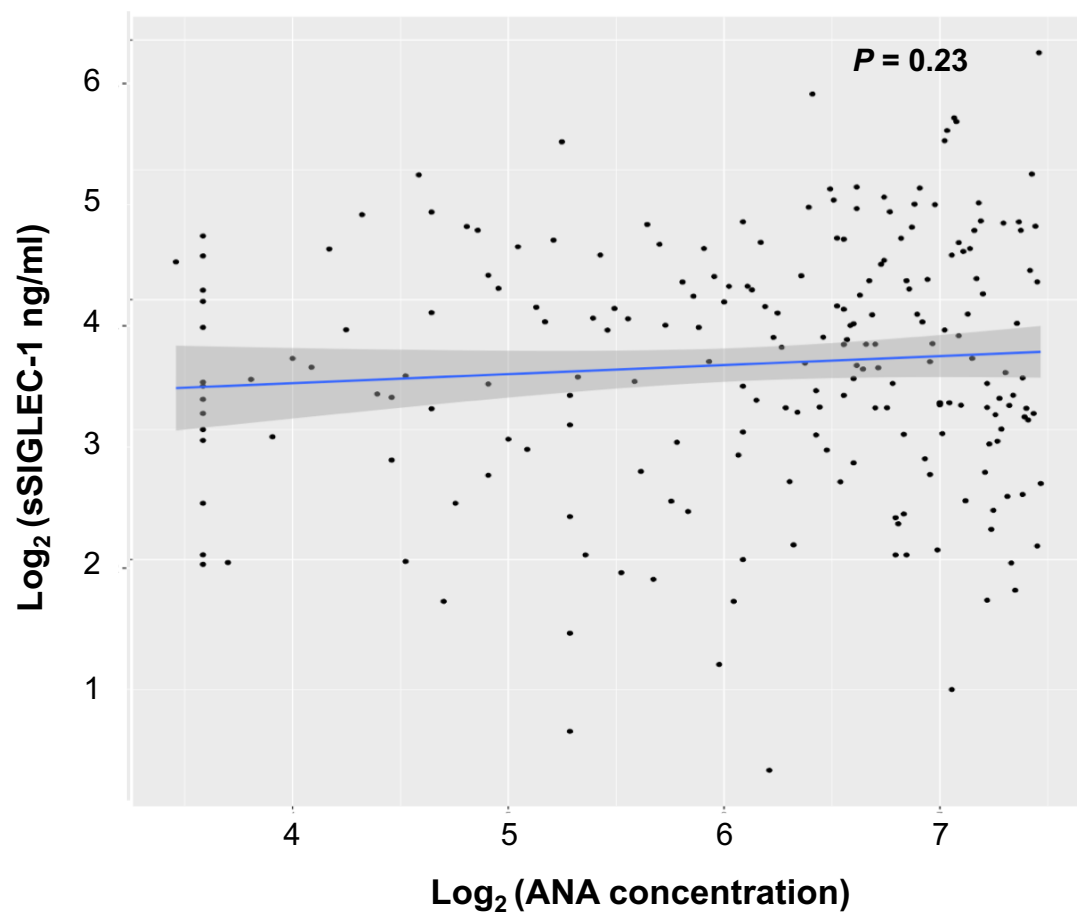

Supplement: Supplementary file 3 — Figure S2. Association of sSIGLEC-1 with serological markers of SLE. a, b Data depict the association of sSIGLEC-1 concentrations with C-reactive protein (CRP) levels (a) and with disease-specific anti-nuclear autoantibody (ANA) titres (b). P values were calculated by linear regression. (PDF 347 kb) [file 13075_2018_1649_MOESM3_ESM.pdf]
